# What is killing? People's knowledge about coronary heart disease, attitude towards prevention and main risk reduction barriers in Ismailia, Egypt (Descriptive cross-sectional study)

**DOI:** 10.11604/pamj.2013.15.137.1628

**Published:** 2013-08-16

**Authors:** Sameh Seef, Anders Jeppsson, Martin Stafström

**Affiliations:** 1 Department of public health, faculty of medicine, Lund University, Sweden; 2 Department of Clinical Sciences, faculty of medicine, Lund University, Sweden

**Keywords:** Non-communicable, coronary heart disease, knowledge, attitude, risk, barriers, Egypt, morbidity, mortality, Public health

## Abstract

**Introduction:**

Cardiovascular diseases are a public health concern everywhere, especially ischemic or coronary heart diseases (CHD) which are on top of causes list of mortality and morbidity in both genders globally. From which nearly 80% can be because of modifiable risks. In Egypt, there is a lack of studies on the knowledge of people about coronary heart diseases and its modifiable risks. So, this research reported here we designed to measure the dimensions of peoples knowledge about CHD and their attitude towards prevention, and to identify the main risk reduction barriers.

**Methods:**

By using comprehensive cross-sectional, descriptive research design, all adult individuals attending the family health clinic at Suez Canal University Hospital were eligible for inclusion with total number 125 participants. An interview questionnaire designed and used to collect data.

**Results:**

The study revealed that (10.4%) of participants had a history of CHD, and (7.2%) had a family history of CHD. 79.2% Had a satisfactory total knowledge about CHD, and (94.4%) had a positive total attitude towards prevention. Risk reduction barriers as a medical setting barriers were (24%), patient related barriers were (22.4%). Community-societal barriers were almost the same as knowledge barriers which were around (16%). At last the systemic-organizational barriers were (9.6%).

**Conclusion:**

The findings settled that, total knowledge about CHD was satisfactory but lower than the level total of attitude. More effort the health system needs to improve the settings and engage patients in their plans and breaking related barriers, with development of health education programs based on needs assessment. Further studies we recommend to explore the reasons and follow up the changes.

## Introduction

Cardiovascular diseases are a public health concern everywhere, especially ischaemic or coronary heart diseases (CHD) which are on top of causes list of mortality and morbidity in both genders globally [1] CHD is an increased issue and a major cause of illness and deaths in the Middle East Region, responsible for 21% of deaths, from which nearly 80% because of modifiable risks [2].

Globally and within the region the sedentary life, high fat diet, high blood pressure, smoking, diabetes, obesity, dyslipidaemia and stress are the main risks which lead to increased prevalence of CHD and especially in Egypt [2] The World health organization data have shown that, CHD is responsible for 10% of Disability adjusted life years (DALY) lost in low- and middle-income countries. In Egypt it is responsible for 21% of fatality and 13% of DALY [3].

The region countries are suffering from a tow fold burden, from both infectious and non-infectious diseases, where shortly the non-infectious diseases will be the most. And as shown from the data that CHD will impose the highest within the cardiovascular diseases and the country′s burden of disease in men and women [4, 5]. It is now an emerging major health problem in low and middle income countries. The incidence curve of CHD, between Egyptians, has risen in the last few decades. This is a general impression among Egyptian doctors repeatedly discussed in scientific meetings.

Presence of the different risk factors affects the CHD mortality, specially high blood pressure, cholesterol level, smoking, physical inactivity, stress and diet. Up to 90% of mortality cases have a risk factor or more because of their different living style [4, 6].

Where in Egypt the major risk factors prevalence are as the following; smoking is nearly 48% for men and 4% for women, hypertension is almost 31% [7]. DM is 7.8% in urban areas, 5.6% in rural agricultural areas for 2.5% in rural desert areas, and obesity is 55.6% [8].

Where is a fairly long time between exposure to a risk factor and development of disease, so there is a need to focus efforts on the risk factors that predict disease. As well the risk factors distribution within the population is needed for planning of prevention.

Health systems need a preventive tactics which focus on the population as a whole, and specifically on the people at high risk of certain diseases. Prevention can be effective but it is usually missed. More than half of deaths because of CHD occurred outside the health facilities for that, the role of primary prevention is increasing and risk factors identification and barriers to risks reduction is getting more valuable [7] Starting a healthy living style by stop smoking, losing weight and starting to be active is the base for prevention and treatment of heart disease [9].

Men, as well as women should be more aware of their own risk of developing CHD and of the manifestations of CHD. Doctors should ask patients more deeply and comprehensively about their illnesses understanding, beliefs, and attitudes to check their knowledge [10], and define the barriers to risk reduction.

In Egypt, there is a lack of studies on the knowledge of people about coronary heart diseases and its modifiable risk factors. So, this research reported here was designed to measure the dimensions of people;s knowledge about CHD and their attitude towards prevention, and to identify the main risk reduction barriers. This study will contribute to the existed knowledge and will add more understanding to the risk factors that we can prevent and control. As well as a step for breaking down the barriers.

This study aimed to identify People's knowledge about coronary heart disease, their attitude towards prevention and main risk reduction barriers. That was achieved through measuring their knowledge and attitude towards CHD, its risk factors and identification of the main barriers for achieving the risks reduction.

## Methods

**Study design:** we used the comprehensive descriptive cross-sectional study design to assess the knowledge, attitude about CHD, the possibly related variables and the main risk reduction barriers.

**Study setting:** This study was carried out in Ismailia governorate, Egypt. It has a population (including surrounding rural areas) of about 750,000, on the west bank of the Suez Canal, nearly halfway between Port Said to the north and Suez to the south.

**Population and fieldwork:** All adult individuals, males and females, attending the family health clinic at Suez Canal university hospital within the inclusion criteria, 18 and older and willing to take part in during the time of the study. On obtaining the official permits, and after preparation of the study tools, the data collection ran for 3 days per week (clinic working days) over 3 weeks duration. The researcher approached eligible individuals according to inclusion criteria, explained to them the purpose of the study, asked their willingness to take part in, and obtained their verbal consent. All participants interviewed using the questionnaire form, and the responses marked by the researcher to avoid any bias in data collection.

**Sampling:** Through a comprehensive random sample within the determined time of the data collection, all individuals fulfilling the inclusion criteria were included: 125 participants, 51 males and 74 females, with no refusal cases for taking part in the interview.

**Data collection tool:** the interview questionnaire we designed based on relevant literature, experts’ opinions and field-testing. It consisted of Personal data (demographic characteristics and socioeconomic data), Medical history, Family history, Knowledge about coronary heart disease, Attitude towards prevention and lastly Barriers for risk reduction. With timing of 10-15 minutes to be marked.

**Scoring:** For the knowledge items scoring was carried out by evaluating the total knowledge through responding to 12 questions asking about the different aspects of knowledge. Disease definition, presentation, main risk factors, fatality and seriousness, early treatment and control, and lastly the main risk factors relative to the incidence of the disease. Right or positive answer was given 2 points and wrong or negative answer was given 1 point. Resulted in a scale for weighting the total knowledge (lowest score was 12 and highest score was 24). Satisfactory level of knowledge chose to be over 75% of total score (>18 points)

In The same principle the total attitude was scored as the following. 1 point was given to the answer (insignificant), and 5 points were given to the answer (very Important), 4, 3 and 2 points were given to the answers (important, average importance, less important) respectively. Answering 5 questions reflecting the attitude aspect Resulted in a scale for weighting the total attitude towards prevention (lowest score was 5 and highest score was 25). Positive attitude chose to be over 75% of total score (>18.75=19 points)

**Ethical considerations and human rights:** A verbal consent was taken from all the studied individuals before the beginning of the study. Complete confidentiality of the data ensured. The investigator provided counseling to participants if needed or requested. Participants had the right to leave and stop the questionnaire.

**Data analysis:** Statistical analysis carried out by using SPSS -V18.0 statistical software packages.

Frequencies and cross tabulations were generated and A chi-square test was used to see if there is a relationship between the categorical variables. Chi-square test, is assuming that the expected value for each cell is five or higher, and statistical significance was considered at p-value <0.05.

## Results

The socio-demographic characteristics of study participants are described in Table 1. It shows that more than 75% were less than 45 years old, with more females (59.2%). More than half of the participants (56%) were either housewives or unemployed, (56.8%) were rural residents, and the majority was married and has children (76.8%). About education, the highest percentage (41.6%) were illiterate and only (6.8%) were highly educated. Only (7.2%) were current smokers. Income was enough for (69.6%) of study participants and not enough for living for (24%).

**Table 1:** Socio demographic data (total n= 125)

Variable	Frequency	%
**Age**		
(18-45)	96	76.8
(46-65)	25	20
(66-75)	4	3.2
**Gender**		
Male	51	40.8
Female	74	59.2
**Occupation**		
Housewife/ unemployed	70	56
Employee	28	22.4
Farmer	27	21.6
**Residency**		
Rural	71	56.8
Urban	54	43.2
**Marital status**		
Married and having children	96	76.8
Married and not having children	20	16
Single	9	7.2
**Education**		
Illiterate	52	41.6
Secondary education	31	24.8
Read and write	22	17.6
Intermediate education	12	9.6
High education	8	6.8
**Special habits (smoking)**		
Non-smoker	116	92.8
Current smoker	9	7.2
**Income**		
Enough	87	69.6
More than enough	8	6.4
Not enough	30	24.0

The socio-demographic data of total 125 participants in the descriptive cross sectional study (people's knowledge about coronary heart disease, their attitude towards prevention and main risk reduction barriers), Ismailia, Egypt, 2010

As shown in Table 2, (10.4%) had a history of CHD, whereas (7.2%) had a family history of CHD. The table also reflected a positive history of other chronic diseases (Diabetes Mellitus, hypertension) among (15.2%) of the participants, and among (36.8%) of their families.

**Table 2:** Medical history (total n= 125)

	Frequency	%
**Personal history of Coronary heart disease**		
No	112	89.6
Yes	13	10.4
**Personal history of Other chronic diseases**		
No	106	84.8
Yes	19	15.2
**Family history of Coronary heart disease**		
No	116	92.8
Yes	9	7.2
**Family history of Other chronic diseases**		
No	79	63.2
Yes	46	36.8

The medical history, data of total 125 participants in the descriptive cross sectional study (people's knowledge about coronary heart disease, their attitude towards prevention and main risk reduction barriers), Ismailia, Egypt, 2010

Table 3 shows the knowledge about CHD among participants. (39.2%) Did not know what the meaning of CHD was. (59.2%) considered the chest pain as disease presentation. (50.4%) defined most of the risk factors. (30.4%) considered smoking as a risk factor and its cessation as a preventive measure (95.2%). Conversely (0.8%) defined diabetes mellitus as a risk factor by itself. Overall (20.8%) of participants had unsatisfactory knowledge about CHD.

**Table 3:** Knowledge aspects (total n= 125)

Knowledge about Coronary heart disease	Frequency	%
**Definition**		
I do not know	49	39.2
Heart arteries disease	37	29.6
Weak heart muscle	26	20.8
Other	13	10.4
**Disease presentation**		
Chest pain	74	59.2
Chest pain with any effort	33	26.4
I do not know	14	11.2
Chest pains disappear at rest	4	3.2
**Risk factors**		
Smoking	38	30.4
Nervousness and stress	8	6.4
High blood pressure	4	3.2
Increased fat intake and Overweight	4	3.2
Inactivity and sedentary life	2	1.6
**Diabetes mellitus**	1	0.8
All risk factors	63	50.4
I do not know	5	4.0
Fatality and seriousness	71	56.8
Early treatment and control importance	107	85.6
* Risk and disease incidence		
Smoking	119	95.2
Overweight	95	76.0
Nervousness and stress	94	75.2
Inactivity and sedentary life	92	73.6
High blood pressure	84	67.2
Diabetes mellitus	73	58.4

The knowledge aspects, data of total 125 participants in the descriptive cross sectional study (people's knowledge about coronary heart disease, their attitude towards prevention and main risk reduction barriers), Ismailia, Egypt, 2010.

* The participants could choose more than one risk factor regarding that question.

About participants’ attitudes towards prevention of CHD, Table 4 shows that most of them had a positive attitude towards all preventive measures. Ranging from (94.4%) for stopping smoking to (73.6%) for the control of diabetes mellitus, with total positive attitude (94.4%).

**Table 4:** Attitude towards prevention

* Preventive measures	Frequency	%
Stopping smoking	118	94.4
Reduction of fat intake and overweight	98	78.4
Control of high blood pressure	97	77.6
Reduction of Nervousness and stress	95	76.0
Control of diabetes mellitus	92	73.6

Attitude towards prevention, data of total 125 participants in the descriptive cross sectional study (people's knowledge about coronary heart disease, their attitude towards prevention and main risk reduction barriers), Ismailia, Egypt, 2010

* The participants could choose more than one preventive measure regarding that question.

The main barriers preventing from achieving risk reduction as in Table 5 considered to be mainly medical setting barriers (24%) followed by patient related barriers (22.4%) and systemic and organizational barriers listed to be the least (9.6%).

**Table 5:** The main barriers preventing from achieving risk factors reduction (total n= 125)

Barriers	Frequency	%
Medical setting barriers	30	24.0
Patient related barriers	28	22.4
Community and societal barriers	21	16.8
Knowledge barriers	20	16.0
Mixed barriers	14	11.2
Systemic and organizational barriers	12	9.6

The main barriers toward prevention, data of total 125 participants in the descriptive cross sectional study (people's knowledge about coronary heart disease, their attitude towards prevention and main risk reduction barriers), Ismailia, Egypt, 2010

The association between the total knowledge and socio-demographic characteristics of study participants as in Table 6 reflected that, there were associations between total knowledge and both of marital status and educational level. And for the associations between the total knowledge and medical and family histories there was only an association with family history of other chronic diseases.

**Table 6:** Association between total knowledge and socio-demographic characteristics of study population

	Total Knowledge			p-value	
	Satisfactory		Unsatisfactory		
	No.	%	No.	%	
**Age (years)**					
(18-45)	80	80.8	16	61.5	
(46-65)	17	17.2	8	30.8	
(66-75)	2	2.0	2	7.7	0.083
**Gender**					
Male	42	42.4	9	34.6	
Female	57	57.6	17	65.4	0.471
**Education**					
Illiterate	34	34.3	18	69.2	
Secondary education	28	28.3	3	11.5	
Read and write	19	19.2	3	11.5	
Intermediate education	12	12.1	0	0.00	
High education	6	6.1	2	7.7	0.015*
**Marital status**					
Married and having children	81	81.8	15	57.7	
Married and not having children	12	12.1	8	30.8	
Single	6	6.1	3	11.5	0.032*
**Job**					
Housewife/ unemployed	54	54.6	16	61.5	
Employee	24	24.2	4	15.4	
Farmer	21	21.2	6	23.1	0.627
**Residency**					
Rural	56	56.6	15	57.7	
Urban	43	43.4	11	42.3	0.918
**Income**					
Enough	69	69.7	18	69.2	
More than enough	6	6.1	2	7.7	
Not enough	24	24.2	6	23.1	0.952
**Special habits ( smoking)**					
Non-smoker	78	78.8	20	76.9	
Current smoker	21	21.2	6	23.1	0.837

* *Statistically significant at p<0.05*. Data of total 125 participants in the descriptive cross sectional study (people's knowledge about coronary heart disease, their attitude towards prevention and main risk reduction barriers), Ismailia, Egypt, 2010

The association between the total attitude and socio-demographic characteristics of study participants reflected the association between total attitude to marital status, job and special habits. But for the association between the total attitudes, medical and family histories there was only an association with family history of chronic heart disease. Also there was an association between the total knowledge and the total attitude.

## Discussion

This study aimed to identify people's knowledge about coronary heart disease, their attitude towards prevention and main risk reduction barriers. In disagreement with the study prevalence findings for CHD and risk factors in participants, an American report in 2003 showed that a large part of the US population had multiple risk factors for heart disease [11]. Moreover, a study in Jordan reported that more than half of the sample had a family history of hypertension and diabetes mellitus [12]. These figures are much higher than those within the present study. The differences between these studies might be because of the different perspective of each study, the targeted population, and the methods of data collection and interpretation.

In studying the knowledge of the studied sample, in the present study, about the risk factors related to CHD, smoking was the most recognized factor by most of the studied participants. Besides, most of them had a positive attitude towards smoking cessation as a preventive measure for CHD. Explained by the increased preventive measures targeting smoking, also spreading awareness among the population all over the country through all mass media Another explanation was the higher number female participants in the present study, who were mostly non-smokers.

About the knowledge of the modifiable risk factors among all participants, the studied factors were recognized by half of the participants. The total knowledge score found to be satisfactory by most of the participants. Moreover, the data showed that most of them had a positive attitude towards all preventive measures. However, the study also reported several barriers to prevention and achieving risk reduction. In this context, surveys of CHD prevention-related services such as: smoking cessation advice, treatment of lipid disorders, physical activity assessment and counseling are disappointing [13]. We should also note that, individuals are expecting a lot from health systems, and less from their own selves? which affect their actions and decisions and so their lives [14].

Knowledge about the disease presentation was correct among most of the participants in the present study. This higher awareness might be because of that chest pain, which is one of the most important symptoms in CHD, and the most feared symptoms. The high-level of awareness about this symptom we quietly expected it, and was in line with the current trends. Patient with a recent onset of chest pain, especially when the symptoms are constant, should be transported immediately to the emergency [15].

Also agreed with the present study findings, the data reported on the signs and symptoms of heart attack and stroke in New York State 2003 had showed that recognition of symptoms ranged from 42% to 93%. Chest pain or discomfort has been the most often recognized symptoms (93%) [16] Further, and as our study finding, data from the 2001 study of the CDC showed that 95% of respondents recognized chest pain as a heart attack symptom [17]. Compared to (88.8%) in the present study.

Although a good percentage of the studied participants recognized the disease presentation, only slightly more than half recognized the seriousness of the disease while most of the participants recognized the importance of early treatment and control. Such inconsistency might lead to the delay in time for treatment. For that It should be kept in mind that knowing the disease presentation is not the only factor that affects the time to treatment, other factors also need to be considered [16].

Within this study, a satisfactory total knowledge level about CHD was revealed among four fifths of the participants. Also, most of them had a positive total attitude. These figures are higher than the similar ones in other studies. Surveys conducted by the AHA between 1997 and 2003 have shown that the awareness of heart disease ranges from 30% in 1997 to 46% in 2003. Excellent awareness was reported by less than half of the population [18].

About the attitude towards CHD, in 1997, a telephone survey of 1000 US households found that only 8% of population respondents identified heart disease as their greatest health concern. The same survey showed that less than one third identified heart disease as the leading cause of death [19]. Another international survey revealed a considerable degree of indifference to coronary heart disease, despite possessing a reasonable knowledge of the risks involved, even among patients who had suffered a myocardial infarction [20]. These findings are in disagreement with the present study results where the attitude was much higher than the knowledge about CHD among the participants.

According to the present study findings, the association between attitude towards prevention among participants, their personal and family histories revealed no association except for the association with the family history of coronary heart disease. This is in disagreement with the claim that, patients who did not experience chest pain during the acute event had significantly different attitudes than those who did [21].

In the present study, obviously the total knowledge score was associated to marital status and educational level. However, it was not associated to age, gender and income level, which in contrast with 2003 results where were people from lower income and certain age groups lag behind the rest in their recognition of these symptoms [16].

The findings are also in line with the results of a study done in two New England communities where knowledge was higher among more educated individuals [22]. Similar findings were reported in three population-based cross-sectional surveys in two northern California cities were conducted between 1980 and 1990 [23], and in Pakistan [24]. Differences or likenesses between these studies might be because of the perspective of each study, the targeted population, and the methods of data collection and interpretation.

In the present study, the relation between participants’ attitude towards prevention and their socio-demographic characteristics pointed that, there was no association between attitude and gender. As well as between age and total attitude, where there was no association. These findings are in contrast with the results of 2005 which had also suggested that educating women and interpreting the symptoms of CHD remain significant obstacles in reducing decision time [15]. Also, older age, female sex, low education level, low socioeconomic status, and black race, were reported to be associated with increased delays in seeking treatment as reported by Moser 2006 [25]. These discrepancies between the results of the previous studies and the current study might be explained by individual characteristics, social, psychological and cultural differences.

The data of the present study showed that, there was an association between knowledge about CHD and attitude towards prevention among participants in the current study. This means that increased knowledge would lead to improved attitude. This is in line with the findings that have emphasized that, interventions based on simple messages, for example knowledge about disease presentations and dealing with it is still being recommended [26]. Lastly, self-reported information subjected to recall and social desirability biases [27] and inability to examine neither the cardiovascular risk factors nor the control of the participants. Besides unawareness of some participants about their risk factors such as high cholesterol, diabetes, or high blood pressure, also Unequal access to the health care services because of any possible obstacles, were the most probably limits of our study.

Finally, this study only examined modifiable risk factors and did not include other fixed risk factors, for example age, gender and family history of coronary heart diseases [9, 17].

## Conclusion

It is concluded that, the level of satisfactory knowledge about CHD and positive attitude towards prevention were higher than expected, but with no statistical significance related to gender or education. There were a highly reported percentage of medical setting related barriers and patient related barriers that were preventing from achieving the risk reduction actions. Therefore, it is recommended to strength the role of doctors in the development and application of health prevention and promotion programs towards CHD and engage patients and families into the risk reduction plans. Further in-depth studies are needed for more accurate results and confirming the findings and cover the limits of the quantitative studies, by using focus group discussions or interviews and using qualitative methods as well.

## References

[1] WHO (2008). The global burden of disease: update 2004. http://www.who.int/healthinfo/global_burden_disease/GBD_report_2004update_full.pdf.

[2] WHO Chronic Diseases and Their Common Risk Factors. http://www.who.int/chp/chronic_disease_report/media/Factsheet1.pdf.

[3] WHO The Atlas of Heart Disease and Stroke, parts 1, 2 and 6. http://www.who.int/cardiovascular_diseases/resources/atlas/en/.

[4] WHO Reducing risks, promoting healthy life. http://www.who.int/whr/2002/en/index.html.

[5] Vasan R.S, Lisa M. Sullivan, Peter W.F. Wilson, Christopher T. Sempos, Johan Sundström, William B. Kannel (2005). Relative Importance of Borderline and Elevated Levels of Coronary Heart Disease Risk Factors. Annals of Internal Medicine.

[6] Topol EJ, Steven PM, Brian PG (2000). Manual of cardiovascular medicine, cardiovascular risk factors.

[7] Mohsen Ibrahim M (2004). Short review of hypertension, coronary artery disease, Egyptian Hypertension Society Guidelines. http://www.ehs-egypt.net/pdf/cor-art-dis.pdf.

[8] Radwan M, Gadslab Shabinaz (2002). Epidemiology of diabetes Mellitus in Egypt. The Egyptian journal of community medicine..

[9] Scott M. Grundy, Gary J. Balady, Michael H. Criqui, Gerald Fletcher, Philip Greenland, Loren F. Hiratzka (1998). Primary Prevention of Coronary Heart Disease: Guidance from Framingham: A Statement for Healthcare Professionals from the AHA Task Force on Risk Reduction, American heart association. Circulation.

[10] WHO (2005). Therapeutic education of patients with coronary heart disease, Europe. http://www.euro.who.int/document/E88278.pdf.

[11] (2005). Racial, Ethnic and Socioeconomic Disparities in Multiple Risk Factors for Heart Disease and Stroke, United States. http://jama.ama-assn.org/cgi/content/full/293/12/1441.

[12] Al-Safi SA, Alkofahi AS, El-Eid HS (2005). Public Response to Chest Pain in Jordan. European Journal of Cardiovascular Nursing..

[13] EUROASPIRE I and II Group; European Action on Secondary Prevention by Intervention to Reduce Events (2001). Clinical reality of coronary prevention guidelines: a comparison of EUROASPIRE I and II in nine countries. EUROASPIRE I and II Group. European Action on Secondary Prevention by Intervention to Reduce Events. Lancet.

[14] Kelly DT (1997). Paul Dudley White, International Lecture. Our future society, a global challenge. Circulation..

[15] Rosenfeld AG, Lindauer A, Darne BG (2005). Understanding Treatment-Seeking Delay in Women with Acute Myocardial Infarction: Descriptions of Decision-Making Patterns. American Journal of Critical Care..

[16] Brian D. Fisher, Margaret Casey, Thomas Melnik (2003). Heart Attack and Stroke: Signs & Symptoms. BRFSS..

[17] Greenlund KJ, Keenan NL, Giles WH, Zheng ZJ, Neff LJ, Croft JB (2004). Public Recognition of Major Signs and Symptoms of Heart Attack: Seventeen States and the US Virgin Islands, 2001. American Heart Journal..

[18] Lori Mosca, Anjanette Ferris, Rosalind Fabunmi, Rose Marie Robertson (2004). Tracking women's Awareness of Heart Disease. An American Heart Association National Study. American Heart Association, Circulation..

[19] Mosca L, Jones WK, King KB, Ouyang P, Redberg RF, Hill MN (2000). Awareness, Perception, and Knowledge of Heart Disease Risk and Prevention among Women in the United States: American Heart Association Women's Heart Disease and Stroke Campaign Task Force. Arch Family Med..

[20] Kurt J. Greenlund, Zhi Jie Zheng, Nora L. Keenan, Wayne H. Giles, Michele L. Casper, George A. Mensah (2004). Trends in Self-Reported Multiple Cardiovascular Disease Risk Factors among Adults in the United States, 1991-1999. Arch Intern Med.

[21] Horne R, James D, Petrie K, Weinman J, Vincent R (2000). Patients’ Interpretation of Symptoms as a Cause of Delay in Reaching Hospital during Acute Myocardial Infarction. Heart..

[22] Kim M. Gans, Susan F. Assmann, Anthony Sallar, Thomas M. Lasater (2002). Knowledge of Cardiovascular Disease Prevention: An Analysis from Two New England Communities 1999. Preventive Med.

[23] Davis SK, Winkleby MA, Farquhar JW (1995). Increasing disparity in knowledge of cardiovascular disease risk factors and risk reduction strategies by socioeconomic status. American journal of Preventive Medicine..

[24] Dodani S, Mistry R, Khwaja A, Farooqi M, Qureshi R, Kazmi K (2005). Prevalence and Awareness of Risk Factors and Behaviours of Coronary Heart Disease in an Urban Population of Karachi, the Largest City of Pakistan: A Community Survey. J Public Health (Oxf).

[25] Moser DK, Kimble LP, Alberts MJ, Alonzo A, Croft JB, Dracup K (2006). Reducing Delay in Seeking Treatment by Patients with Acute Coronary Syndrome and Stroke: A Scientific Statement from the American Heart Association Council on Cardiovascular Nursing and Stroke Council. Circulation..

[26] (2000). National service framework for coronary heart disease: Department of Health, England. http://www.dh.gov.uk/en/Publicationsandstatistics/Publications/PublicationsPolicyAndGuidance/DH_4094275.

[27] Hugh J (1981). Arnold and Daniel C.Feldman. Academy of Management Journal..

